# Identifying barriers and facilitators for implementing harm reduction strategies for methamphetamine use into hospital settings

**DOI:** 10.3389/frhs.2023.1113891

**Published:** 2023-02-07

**Authors:** Cheryl Forchuk, Jonathan Serrato, Leanne Scott

**Affiliations:** ^1^ Mental Health Nursing Research Alliance, Lawson Health Research Institute, London, ON, Canada; ^2^ Arthur Labatt Family School of Nursing, Western University, London, ON, Canada

**Keywords:** harm reduction, hospitals, substance use, nursing, health equity, qualitative research

## Abstract

**Introduction:**

Harm reduction strategies for substance use disorder are not currently offered in Canadian hospitals. Previous research has suggested that substance use may continue to occur which can lead to further complications such as new infections. Harm reduction strategies may be a solution to this issue. This secondary analysis aims to explore the current barriers and potential facilitators for implementing harm reduction into the hospital from the perspective of health care and service providers.

**Method:**

Primary data was collected from 31 health care and service providers who participated in a series of virtual focus groups and one-to-one interviews regarding their perspectives on harm reduction. All staff were recruited from hospitals in Southwestern Ontario, Canada from February 2021 to December 2021. Health care and service professionals completed a one-time individual interview or a virtual focus group using an open-ended qualitative interview survey. Qualitative data was transcribed verbatim and analyzed using an ethnographic thematic approach. Themes and subthemes were identified and coded based on responses.

**Findings:**

Attitude and Knowledge, Pragmatics, and Safety/Reduction of Harm were identified as the core themes. Attitudinal barriers such as stigma and lack of acceptance were reported but education, openness and community support were regarded as potential facilitators. Cost, space, time and availability of substances on site were regarded as Pragmatic barriers but potential facilitators such as organizational support, flexible harm reduction services and a specialized team were identified. Policy and liability were perceived as both a barrier and a potential facilitator. Safety and impact of substances on treatment were considered as both a barrier and a potential facilitator but sharps boxes and continuity of care were regarded as potential facilitators.

**Discussion:**

Although barriers in implementing harm reduction in hospital settings exist, there are opportunities to facilitate change. As identified in this study, feasible and achievable solutions are available. Education on harm reduction for staff was considered to be a key clinical implication in facilitating harm reduction implementation.

## 1. Introduction

Methamphetamine known also as meth, ice and crystal is a highly addictive stimulant with chronic use potentially leading to violent behaviour and psychotic symptoms such as paranoia, delusions and hallucinations (1, 2). The substance has also been found to have harmful physical consequences such as cardiotoxicity, respiratory failure and seizures (3) as well as negative effects on the immune system (4). Further, health-compromising behaviours including sharing needles and paraphernalia come with dangerous consequences such as HIV and other infections (5, 6). In hospital, it has been found that the risk of infection is higher among people who inject substances compared to those in outpatient community settings (7). A potential way of addressing these issues and improving safety for patients and staff may be through utilizing a harm reduction approach. The purpose of this paper is to observe the current barriers and potential facilitators that may exist in implementing harm reduction strategies into the hospital setting.

While establishing harm reduction practices in hospital may be beneficial, it is faced by many barriers. Previous research that investigated the needs of frequent users of the emergency department (ED) with addiction issues highlighted stigma by health care professionals as a barrier to care (8, 9). Another study exploring attitudes of nurses caring for opioid use disorder identified several barriers in establishing harm reduction including stigma, perceptions of drug-seeking behaviour, burnout, treating pain, safety, and communication (or lack thereof) between health care professionals (10). As well, a lack of understanding as to what harm reduction entails can also provide a challenge to implementation among health care professionals (11). Additional education and training may therefore be required. Education, even among substance use treatment professionals, has been found to increase openness and change attitudes towards harm reduction (12).

Another barrier can be loss of empathy which has been found to lead to negative interactions with people who use substances (13) Aggressive behaviour towards staff as a result of methamphetamine use has been reported in numerous studies (10, 14, 15) and could result in difficulty for health care staff to empathize when under duress. Aggression, social cognition and recognising facial expressions have been found to be impaired among individuals who use methamphetamine (16–18). Delusions, paranoia or hallucinations as a result of long-term use may also result in aggression (19). Community pharmacists maintain that lack of training and fear of attracting disruptive clientele are safety barriers to implementing harm reduction strategies (20).

Despite these barriers, there have been a small number of studies that have investigated facilitators that can help with establishing harm reduction practices. Physicians with addiction training and an addiction-related research portfolio, and access to community-based treatment upon discharge, have been found to enhance readiness for addiction consultation services in hospital (21). This was supported by a study that found community-based efforts to address overdose can reduce harms associated with substance use (10). In terms of resources and funding, combining infection treatments with harm reduction services such as substance substitution has been found to be more cost-effective in comparison to partial approaches such as needle programs or opioid substitution therapy alone (22). A literature review by Wilson et al. (23) on various combined harm reduction strategies and a study by Ciketic et al. (24) into counselling for methamphetamine use both reported a high degree of cost-effectiveness.

There is a need for harm reduction strategies for patients who use methamphetamine but this can be fraught with a number of challenges despite their benefits. As part of a larger project aiming to develop and introduce such strategies, it was necessary to evaluate the current issues from a health care provider perspective and understand what needs to be addressed. This secondary analysis of the current study set out to explore the current barriers and potential facilitators to the implementation of harm reduction strategies in hospital settings.

## 2. Method

### 2.1. Design

The first year of this four-year study set out to explore experiences, recommendations and considerations for harm reduction strategies to be implemented into hospital settings. This particular paper is focusing on the issue of barriers and potential facilitators raised by health care and service professionals. Health care and service professionals completed either an individual interview or a virtual focus group using an open-ended qualitative interview survey. Recruitment for this study began in February 2021 and concluded in December 2021. It was anticipated that these findings would inform the type of harm reduction strategies to be developed as well as areas of concern to be addressed. This study received ethical approval from the Western University Research Ethics Board and Lawson Health Research Institute.

### 2.2. Sample

Health care and service professionals were recruited using a convenience sampling approach *via* word-of-mouth and email bursts from the study's Advisory Group which included physicians and other nursing staff as group members. Individuals who received emails were invited and also asked to forward the email to other colleagues. Other service professionals were contacted by members of the Advisory Group to circulate information about the focus groups. Word-of-mouth information regarding the focus groups was also presented at Advisory Group meetings and the study's subcommittee meetings to promote recruitment and encourage dissemination. Subcommittees included an Education subcommittee consisting of nurse educators and physicians, a Leadership subcommittee consisting of senior hospital management, and a Research subcommittee focusing on knowledge translation consisting of researchers. Staff from all four participating hospitals in Southwestern Ontario, Canada were invited to participate. Interested individuals then contacted the Research Coordinator, Principal Investigator or a member of the Advisory Group to arrange a time and day convenient for them. Those who offered to participate in the focus groups and one-to-one discussions were health care and service professionals who directly provided care and related services to individuals using methamphetamine. The study was open to all health care and service professionals with differing opinions towards harm reduction principles in order to develop a richer understanding of the spectrum of opinions and beliefs.

### 2.3. Procedure

Health care and service professionals were asked to participate in a virtual setting with focus groups or one-to-one discussions conducted *via* teleconferencing software due to the COVID-19 pandemic. Focus groups/discussions were held with two research team members; one to facilitate the discussion and another to take notes to track non-verbal communication as well as distinguish between speakers to ensure the quotes used reflected the correct individuals. All health care and service professionals provided informed consent before any one-to-one interviews or focus groups took place. During the discussion, a semi-structured interview guide of questions to gather information and promote discourse was followed. This included barriers and facilitators to implementing harm reduction as well as the perceived current issues of no harm reduction and recommendations for change or not to change. The questions focused on the hospital setting as a whole such as organizational issues and current operations as well as personal opinions or beliefs. The interview guide contained the following questions:

(1) What are some of the issues with the current approach to harm reduction strategies and methamphetamine use in the hospital?
(2) What do you think should be changed within the current approaches to harm reduction?
(3) What are some aspects you would not change within the current approaches to harm reduction?
(4) What would be some of the facilitators in implementing a new approach to reducing harm for methamphetamine use in the hospital?
(5) What would be some of the barriers in implementing a new approach to reducing harm for methamphetamine use in the hospital?

The interviews and focus groups were recorded using an audio recorder to ensure all comments, suggestions and discussions could be transcribed for analyses. All focus groups and discussions were audio recorded and transcripts were written verbatim by trained research assistants. Health care and service professionals were offered a $5 coffee shop gift card upon completion.

### 2.4. Analyses

The audio recordings from the interviews and focus groups were securely uploaded to the research team's shared desktop space which can only be accessed by authorized research team members. To ensure the integrity of the transcript and confirm de-identification of the recordings, the transcripts were validated by another research team member. The transcripts were analyzed using a thematic ethnographic approach (25). Themes and subthemes, as well as patterns within each, were identified and coded based on responses. This method of analysis also explicitly examined the cultural context of the group as a whole as well as the individual experiences disclosed. The analyses were conducted by the three authors and identified themes were developed collaboratively and with the consensus of all three authors, one of which was the principal investigator, to ensure the trustworthiness of the findings. All three authors were highly experienced in qualitative analysis and had previously conducted research into substance use, nursing and homelessness. The authors had backgrounds in psychiatric nursing, psychology and nursing respectively, making them knowledgeable in answering the research question.

## 3. Findings

A total of 31 health care and service professionals participated in virtual focus groups and individual discussions. Twenty-six were registered nurses, of which three specialized in public health nursing and five in mental health nursing. The remaining five health care and service professionals included a nurse practitioner, a harm reduction manager, an epidemiologist, a social worker and a housing services manager. Three of the four hospitals in Southwestern Ontario were represented.

### 3.1. Themes

Health care and service professionals were asked about the current barriers and potential facilitators for improvement should harm reduction strategies be considered for implementation in hospital settings. Upon analysis of the responses, it became apparent that three larger areas of consideration were based around: 1) attitude and knowledge; 2) pragmatics; and 3) safety and reducing harm. Each area of consideration contained subthemes with some placing as both a barrier and facilitator (see Table 1).

**Table 1:** Themes and subthemes of identified barriers and potential facilitators.

Barriers	Barriers and Facilitators	Potential Facilitators
Attitude and Knowledge		
Stigma Lack of Acceptance	Trust	Education Openness to Harm Reduction Community Support and Acceptance
Pragmatics		
Cost, Space and Time Availability of Substances	Policy and Liability	Organizational Support Flexible Drop-in and Specialized Team
Safety and Reduction of Harm		
	Safety to Staff and Patients Impact on Treatment	Sharps Boxes Implementing Community Support and Continuity

## 4. Theme 1: attitude and knowledge

### 4.1. Barriers

#### 4.1.1. Stigma

Most of the health care and service professionals highlighted that there are issues regarding stigma towards individuals who use methamphetamine. In particular, drug-seeking behavior (defined as a range of behaviours or activities with the goal of attaining drugs (26) and the risk of a patient coming to hospital to acquire substances was discussed by the health care and service professionals. As well, the conduct of individuals who may be under the influence of methamphetamine and/or experiencing withdrawal can lead to negative interactions with health care and service professionals:

*I think it does go back to stigma. I think there will be some people who, based on previous experiences or just prejudices um will have difficulty with a new harm reduction approach. Um I think some, staff have had those bad experiences and often, share those experiences with others um about conduct of people who inject drugs um and specific patients' actions. So, I think it would really be um a barrier in that attitude, like way of thinking… I think that's often like the thought of staff is that um this system is being taken advantage of with the people who inject drugs*.

#### 4.1.2. Lack of acceptance

It was also discussed by almost all of the health care and service providers that there may be pushback and disapproval as the working landscape shifts to a more unfamiliar view. Following from the concept of stigma, encouraging other health care and service professionals and the public to be accepting of a harm reduction approach to care may be met with disagreement.

*You have people that would love to work with, uh, individuals that are using methamphetamine to try to, try and not necessarily get them to stop, but to help them in any way that we can and to help them help themselves. Whereas there are other people that um, would want to have nothing to do with them, um, which is sad, but kind of the harsh reality*.

Of these, a few of the health care and service providers discussed the potential disagreement from members of the public amid concerns around personal safety.

*Again I shouldn't make a generalization, but some people have strongly opposed safe injection sites because they, they don't want them in their neighborhoods or they think that it'll, they attract, they attract people that they might not want around their homes or their kids or whatever it is…*

### 4.2. Potential facilitators

#### 4.2.1. Education

Education was identified as a facilitator to potentially reverse stigma and encourage acceptance among staff. As discussed by most of the health care and service professionals, providing educational resources and additional training on what harm reduction actually is and how it can be beneficial to patients who use methamphetamine may help in generating interest. Creating discourse and enhancing understandings of harm reduction could lead to more individuals being open to the idea of implementation in hospital settings and generate a greater sense of optimism towards it. Further, it was also suggested that peer supporters could inform education for staff by offering a lived experience platform.

*So, if they think harm reduction is great then you're in, if they think harm reduction is not so great then that could be a huge barrier. So, really trying to um convince them is not the right word, but educate them so that they understand why it's so important I think is really, really key*.

#### 4.2.2. Openness to harm reduction

A few health care and service professionals pointed out that greater openness to the idea of harm reduction and greater understandings of addiction would also support successful implementation. As well, it was noted that there is a degree of openness to the concept of harm reduction currently and this could further cultivate changes in practice as a whole.

*Um, I think, I think people are starting to, in terms of, substance use in general, I think people are starting to open up more to the idea of, um, substance use being… an actual diagnosis*.

#### 4.2.3. Community support

Not only was there discussion regarding staff acceptance and openness, but it was also discussed by a few health care and service providers how community agencies can generate support for the provision of harm reduction strategies. Potential examples of community agencies could be those that serve people experiencing homelessness and those that offer harm reduction services in the community. It was stated how they can facilitate the therapeutic relationship between patients and hospital staff and help to improve any trust issues that may be present.

*So I do think that community partners play a huge role in um supporting the word to get out there and supporting um building that trust between um folks that experience homelessness and use crystal methamphetamines and hospital staff*.

### 4.3. Barriers & potential facilitators

#### 4.3.1. Trust

Trust was an issue discussed by many of the health care and service providers. Previous negative experiences between patients and health care staff can lead to a lack of trust between the two groups thus creating a barrier for both sides.

*But um yeah I honestly think that the biggest, so I, I think like the barriers are simply lack of trust in, in the system, that they've failed individuals time and again um yeah, I think that's, that's probably the biggest one*.

In other instances, it was reported by a couple health care and service providers that building therapeutic relationships can enhance a sense of trust and communication which could allay trust issues and encourage uptake of harm reduction strategies. In this case, the role of trust can act as both a barrier and a facilitator.

*I know from talking to people who use our safe injection sites already and from talking to, from talking to those, the staff members who work there, um there's a, there's a really nice sense of community and um, the clients are very, once they get to know the staff they're very trusting of the staff… so it would be really great if we, harm reduction in the hospital and then patients feel that positivity feel that they are supported feel that people want to help them…*

## 5. Theme 2: pragmatics

### 5.1. Barriers

#### 5.1.1. Availability of substances

In terms of accessibility to substances within the hospital, a few health care and service professionals highlighted this could pose a barrier to implementing harm reduction strategies. The difficulty of having methamphetamine on-site and how patients would obtain it could be problematic to their care or may involve an illicit drug dealer coming in to the hospital site.

*And then another thought of mine is how are they, if they're in the hospital and they didn't come in with substances, how are they going to get them?*

*Yeah. They're either going to need to drop them off, get them dropped off or go pick them up*.

#### 5.1.2. Cost, space and time

There are also a number of pragmatic barriers from a financial standpoint that were discussed by most of the health care and service providers. Allocating budgetary expenses to the strategies could prove challenging, particularly in regards to staffing and the potential for additional personnel. As well, resources such as providing an available space to offer the services, plus any equipment needed, could also be a barrier. Also noted frequently in terms of pragmatics were time constraints (i.e., staff workloads, “access and flow” of patients) and logistic constraints (i.e., needing porters to help take patients to harm reduction services on offer) which could also add to future costs. Regarding the latter, one individual suggested it would be advisable for harm reduction services to be brought to the patient as opposed to the patient attending the intervention.

*Does, does a hospital have capacity to have a separate room, and have devoted staff, and have the materials so that people can use while they're in hospital without exposing others to harm?*

### 5.2. Potential facilitators

#### 5.2.1. Organizational support

In order to obtain the necessary funds allocated, several of the health care and service providers noted that there needs to be support and interest from senior management and leadership. There was discussion regarding this requirement as a facilitator financially but health care and service professionals also noted that involvement from those in leadership positions would set an example to follow and could encourage staff acceptance. A couple other health care and service providers proposed further education for senior staff and those in leadership positions to enhance support.

*I think is really key, because in order for staff to be on board, leaders have to be on board*.

#### 5.2.2. Specialized team and flexible service

A potential facilitator identified by several of the health care and service providers was the need for a specialized team with experience in harm reduction and addiction. Providing care for patients with addiction needs can prove to be challenging for frontline staff without the necessary experience. It was suggested they would have specific roles to fulfill such as responding to overdoses or aggressive behaviours, and administering medications such as Naloxone.

*So like a team, a team approach is, is good when there's like a specific team that that's their role. Um, like a harm reduction team. In the hospital*.

In terms of service delivery, another potential facilitator noted by a few of the health care and service providers was the need for flexibility in allowing patients to attend harm reduction services. A service provided at times convenient for them as opposed to appointments which can prove difficult for this population was recommended:

*Drop in hours rather than strict appointment times for patients wanting to attend the consumption site/service, example, Mondays and Fridays and mornings aren't ideal. Those would be facilitators*.

### 5.3. Barriers and potential facilitators

#### 5.3.1. Policy and liability

In order to provide harm reduction strategies, the hospital would require an exemption under the current legislation. Such an exemption could therefore act as a facilitator to harm reduction and provide frontline staff with protection from liability to perform care. Many of the health care and service professionals highlighted their concern for blame and liability should an adverse event occur, particularly in the event using methamphetamine interferes with current treatment and medications.

*Yeah, so if we can absolve the hospital of any type of liability for implementing a service like that, then I think organizational support would no longer be a barrier*.

## 6. Theme 3: safety and reduction of harm

### 6.1. Potential facilitators

#### 6.1.1. Sharps boxes

Several of the health care and service professionals reported that current practice involves removing sharps boxes from the rooms of patients who use substances intravenously. As noted, this can lead to greater risk of needlestick injury. It was also highlighted that sharps boxes were not distributed unless it was for medical purposes.

*Um we remove sharps containers from within the room. Um when we have patients who inject drugs, which I think actually leads to an increased likelihood of like needle incidents like sharp incidents for staff and obviously they can't dispose of their sharps as well. Uh so I think that that's a little counterproductive*.

#### 6.1.2. Implementing community support and continuity

It was briefly discussed by a few of the health care and service providers that continuing safe use beyond discharge would also be beneficial in maintaining harm reduction. One specific recommendation was to have an assigned individual coordinating discharges for patients to be connected with resources in the community and support staff who may not be knowledgeable on such programs.

*So it's just, if you're going to do harm reduction I just think you really have to look at the whole picture from admission to discharge to transition into the community*.

From these responses on this issue, the concept of continuity of care and support in the community also fit into the notion of facilitating safe use and reducing harm after discharge. Providing patients with the tools needed can help them continue to remain safe when back in the community.

*It would be great to be able to just refer patients from the hospital to whatever centre so that they can just kind of run with it when they get out into the community… Um I know that there's uh a tent somewhere in (city) I've never seen it before, a safe injection site*.

### 6.2. Barriers & potential facilitators

#### 6.2.1. Safety to patients and staff

Safety was a much-discussed topic for many reasons, both as a barrier and as a facilitator, by most of the health care and service providers. As a barrier, it was mentioned frequently that individuals under the influence or withdrawing from methamphetamine can become aggressive which can lead to risks to physical safety among frontline staff. Mental health issues such as paranoia and psychotic disorders can be exacerbated leading to confrontational behaviour. Feeling unsafe was a fundamental concern across many health care and service professionals and many reported negative, even violent, experiences with patients of this population.

*People that are using it can sometimes become more agitated or paranoid, which can lead to further dangers in the, in the clinical setting with other patients or with other staff members. Um, and that's ultimately, I guess, kind of the, the risk/benefit of it all*.

It was also discussed by a few of the health care and service providers that the safety benefits of harm reduction strategies could facilitate their introduction. The health care providers suggested that harm reduction in hospital could encourage patients to stay and allow for monitoring of vital signs as well as recovery. Infection rates and negative health outcomes could also be reduced. From a staff safety perspective, it was suggested that additional security or working with an additional member of staff could help provide the level of safety needed to provide care comfortably.

*In terms of positives, I would hope that we would see a lot less infection related to drug injection in the hospital, less negative outcomes in general associated with drug use in the hospital because people have a safe place to go*.

#### 6.2.2. Impact on treatment

Several of the health care and service providers discussed the impact of substance use on treatment in the hospital setting. Safe usage of methamphetamine in hospital was seen as a potential barrier for harm reduction due to the risk of the substance interfering with other medications or impacting medical procedures. However, with the introduction of a harm reduction approach, patients may feel more able to openly discuss their usage with their health care providers and therefore facilitate the development of a safer treatment plan. This means that potential adverse reactions and the interference or delaying of procedures is minimized.

…*like it would be an ideal situation where we could have them use under our care, but sometimes that's just not possible due to the treatment regimen that they're on, so if they do leave and we don't know how to handle that situation or how to talk to them and maybe try to convince them to stay, then yeah it just opens them up to possible harms down the line, right?*

## 7. Discussion

The findings revealed a number of barriers and potential facilitators that can be grouped based on attitude, pragmatics and safety. Barriers included stigma, lack of acceptance, cost, space, time and the availability of substances on-site. Potential facilitators included education, openness, community support, organizational support from hospital leadership, provision of sharps boxes, a flexible and specialized support service within the hospital, and continuity of care into the community. Some issues were both a barrier and a facilitator such as trust (enhanced trust or lack thereof), policy and legislation (current policy is restrictive but policy change can support harm reduction), safety to staff patients (can be heightened but concerns may still exist) and impact on treatment (openness can maintain safety but failure to disclose substance use could affect treatment).

Although there is literature regarding barriers and facilitators to accessing harm reduction for substance use disorder, there is currently a lack of literature examining the introduction of such strategies in hospital settings. A key concept uncovered pertained to attitude and knowledge where issues such as stigma and openness towards harm reduction were discussed. Gaining acceptance from frontline staff could be challenging, particularly as current policy and legislation focus on substance supply and demand reduction which may lead to health care providers feeling contradictory, immoral, or enabling (27, 28). Canadian legislation on harm reduction at the provincial level has only been identified in two provinces (Alberta (29) and British Columbia (30) while one province (Manitoba (31) has regional-level policy (32). Hyshka et al. (32) report that the majority of policies focused on mental health and addiction, infections and substance use only while few recognised stigma and the need for harm reduction. To aid this, community support can act as the bridge between the streets and the hospital and enhance trust in order to facilitate help-seeking as well as maintaining that continuity of safety when back in the community. Involving community members in decision making processes can provide greater understanding on substance use at the local level and attempt to promote openness within the community. Although the concept is still within its infancy, harm reduction strategies on hospital grounds have begun to start up in Canada. A supervised consumption service (33) and a bedside needle/syringe program (34) in Edmonton as well as overdose prevention programs in Vancouver (35) and Victoria (36) have been implemented. Free naloxone kits have also been made available to patients at two hospital emergency departments in Toronto (37).

A common concern in the literature is the change in culture and pragmatics in regards to offering a different type of care that may not be perceived as following conventional treatment. It has been argued that harm reduction represents a pragmatic approach in itself rather than one simply of liberal humanism (38). Improving safety could also enhance trust and therapeutic relationships. Previous qualitative research has revealed that practitioners have responded favorably to harm reduction strategies due to their pragmatism in treating patients with comorbidities through greater engagement with patients and client-centered, achievable goal-setting (27). However, the need for a specialized team and patient concerns around trust have been previously reported by various medical staff and patients (39). There may also be discrepancies as the ED is focused on minimal follow-up whereas patients who use methamphetamine may require long-term care and support after discharge (40, 41), therefore staff must be knowledgeable of resources for referral.

Although harm reduction reduces potential harms for patients, it must also ensure a safe environment for all others in the vicinity. Aggressive or threatening behaviour was discussed during the course of the study and has been highlighted in previous literature among nurses (42) and hospital security staff (43). Physical protection for both staff and patients would include an increased provision of sharps boxes to prevent accidental injury from discarded needles. Previous research has reported a decrease in needlestick injuries for housekeeping and health care staff when patients have had greater access to sharps boxes in the hospital setting (44). A facilitator for ensuring safety would include the provision of sharps boxes which has been found to significantly decrease disposal and needle-recapping injuries (45). This would also reduce transportation-related injuries of syringes from medication stations.

The clinical implications for these findings are widespread. This study highlighted the need for the development of courses that educate health care professionals in substance use and addiction to reduce stigma within the field and facilitate openness. Providing additional education on substance use to nursing students has been found to improve attitudes towards care and intervention towards people who use substances (46). Education and resources including pamphlets on safe injection sites, harm reduction strategies and trauma-informed care for the public have also **been** found to increase acceptability for safe injection sites in the community (47). Linked to this was the suggestion of having trained personnel to provide support for patients as well as staff. Having a specialized team may help to facilitate safety by building therapeutic relationships and supporting adverse reactions, particularly for staff who have not received comprehensive training in the field of substance use. It has been suggested that a singular syringe exchange harm reduction service may not be cost-effective (48), therefore combined services may be preferable. This would allow for greater convenience and flexibility for patients and so it would be recommended that a range of options be provided in one location. Although recent research has reported slightly higher cost savings with sterile syringe programs than syringe and medication combined services, a combined approach would target both medical and non-medical costs (49). More importantly, both conditions were significantly more cost effective than no intervention at all (49). These findings indicate the importance of programs such as sterile supplies and needle exchange. Ultimately, hospital policy will need to be adjusted to reflect harm reduction principles and relieve frontline staff of liability concerns. Current practices such as removing sharps boxes would also need to be revised.

### 7.1. Limitations

This study was conducted largely in one city in Southwestern Ontario in Canada and mostly enrolled health care and service professionals from two hospitals. As well, 26 of the 31 participants were registered nurses and so there would be a need for a greater diversity of staff roles such as physicians, therapists, psychiatrists, etc. Future research in different locations around the country with a wider range of health care and service professionals should be conducted to explore other experiences and generate further understandings of how best to implement harm reduction principles in hospital. Another potential limitation could pertain to self-selection bias as health care and service professionals volunteered to share their opinions on harm reduction in the hospital setting. This may have led to individuals with particularly strong beliefs enrolling themselves to discuss the topic under study. Future research should attempt to recruit health care and service professionals by approaching these individuals directly or randomly selecting groups of staff members. Due to the nature and demands of the COVID-19 pandemic however, it was difficult to enroll individuals in large groups at pre-decided times and dates on a non-volunteering basis.

## 8. Conclusion

The findings from this qualitative analysis suggest that despite the identified barriers in hospital settings, there are also opportunities to facilitate change. Changes in attitude, knowledge, pragmatism and approaches to safety are needed to support the introduction of harm reduction. Policy change and education can provide a useful foundation to initiate this shift in practices and ensure safety for patients during and after hospital care. Future research could seek to explore these issues further by ascertaining barriers and potential facilitators in different locations. Although this would represent a fledgling concept in hospital care, the issues presented and reported suggest that there is room for further investigation. Further research may be able to better inform future treatment and care, even if only small, incremental changes are introduced over time.

## Data Availability

The datasets presented in this article are not readily available. We do not have permission from our Funder or our Ethics Board to share our data freely. Our agreements with both institutions state that data can only be shared with co-investigators/partners for analysis purposes. This was also stated in our Consent forms with participants. We would be open to collaborating on future papers with other research teams where we could share data as partners and add them to our agreements with the Funder and Ethics Board. Requests to access the datasets should be directed to; Jonathan Serrato, jonathan.serrato@lhsc.on.ca.
